# Exploring the Antifungal Activity of Moroccan Bacterial and Fungal Isolates and a Strobilurin Fungicide in the Control of *Cladosporium fulvum*, the Causal Agent of Tomato Leaf Mold Disease

**DOI:** 10.3390/plants13162213

**Published:** 2024-08-09

**Authors:** Zineb Belabess, Bilale Gajjout, Ikram Legrifi, Essaid Ait Barka, Rachid Lahlali

**Affiliations:** 1 Phytopathology Unit, Department of Plant Protection, Ecole Nationale d’Agriculture de Meknès, Km10, Rte Haj Kaddour, BP S/40, Meknes 50001, Morocco; zineb.belabess@inra.ma (Z.B.); bgajjout@enameknes.ac.ma (B.G.); ikramlegr@gmail.com (I.L.); 2 Plant Protection Laboratory, Regional Center of Agricultural Research of Meknes, National Institute of Agricultural Research, Km 13, Route Haj Kaddour, BP.578, Meknes 50000, Morocco; 3 Laboratory of Functional Ecology and Environmental Engineering, Sidi Mohamed Ben Abdellah University, Route d’Imouzzer, P.O. Box 2202, Fez 30000, Morocco; 4 Induced Resistance and Plant Bio-Protection Unit-EA 4707-USC INRAE1488, Reims Champagne-Ardenne University, 51100 Reims, France; ea.barka@univ-reims.fr

**Keywords:** *Solanum lycopersicum*, *Cladosporium fulvum*, biocontrol agents, strobilurins, IPM, FTIR

## Abstract

The causal agent of tomato leaf mold, *Cladosporium fulvum*, is prevalent in greenhouses worldwide, especially under high humidity conditions. Despite its economic impact, studies on antifungal agents targeting *C. fulvum* remain limited. This study evaluates biocontrol agents (BCAs) as alternatives to chemical controls for managing this disease, alongside the strobilurin fungicide azoxystrobin. From a Moroccan collection of potential BCAs, five bacterial isolates (*Alcaligenes faecalis* ACBC1, *Pantoea agglomerans* ACBC2, ACBP1, ACBP2, and *Bacillus amyloliquefaciens* SF14) and three fungal isolates (*Trichoderma* spp. OT1, AT2, and BT3) were selected and tested. The in vitro results demonstrated that *P. agglomerans* isolates reduced mycelial growth by over 60% at 12 days post-inoculation (dpi), while *Trichoderma* isolates achieved 100% inhibition in just 5 dpi. All bacterial isolates produced volatile organic compounds (VOCs) with mycelial inhibition rates ranging from 38.8% to 57.4%. Likewise, bacterial cell-free filtrates significantly inhibited the pathogen’s mycelial growth. Greenhouse tests validated these findings, showing that all the tested isolates were effective in reducing disease incidence and severity. Azoxystrobin effectively impeded *C. fulvum* growth, particularly in protective treatments. Fourier transform infrared spectroscopy (FTIR) analysis revealed significant biochemical changes in the treated plants, indicating fungal activity. This study provides valuable insights into the efficacy of these BCAs and azoxystrobin, contributing to integrated management strategies for tomato leaf mold disease.

## 1. Introduction

Tomato (*Solanum lycopersicum* L.) is one of the most important vegetables worldwide [1,2,3,4,5]. It is the second-most consumed vegetable in the world, either fresh or processed, after potatoes [6]. In 2021, global tomato production was roughly 189 million tons over 516,738.8 ha of cultivated area, with China contributing to 36% of this total production. India, Turkey, and the United States followed, in that order [7]. The tomato crop in Morocco is one of the most important, with a significant socio-economic impact, given that the tomato industry creates thousands of jobs [8]. In 2021, Morocco’s tomato production reached 1.31 million kilograms, cultivated over an estimated 13,875 ha of fresh fruit fields. The yield was 9.44 kg/m^2^, positioning Morocco as one of the world’s top 20 largest tomato producers [9]. Indeed, tomato exports play a significant role economically because they generate roughly 10.411 million DH in foreign currency. Morocco exported 740,660 tons of tomatoes in 2022. This enabled Morocco to rank among the top exporters of tomatoes, rising to take the third position in the same year [10]. 

Numerous biotic and abiotic stresses that may arise separately or in combination frequently limit tomato cultivations [11]. Like most crops, the tomato is vulnerable to various diseases and is prey to a variety of insects [2,4]. While not all threats result in significant losses, a few do and must be addressed for profitable cultivation. Tomato diseases can be caused by fungal, bacterial, viral, or nematode infections, as well as unfavorable environmental conditions [12]. One of the most severe diseases that affect tomato production is tomato leaf mold, caused by *Cladosporium fulvum* Cooke (previously known as *Fulvia fulva* and *Passalora fulva*) [3,13,14,15,16,17]. *C. fulvum* is a highly specialized plant pathogen used as a model for functional studies on plant pathogenic *Mycosphaerellaceae* [18]. It is widespread among tomato-growing countries and is a major economic concern [19], affecting plant development, fruit quality, and yield [20]. 

*C. fulvum* primarily infects leaves but can also spread to stems and fruits, typically occurring in the middle and late stages of production [21]. The most noticeable symptoms are white mold spots or patches that turn brown upon sporulation on the abaxial side of the leaf [18,19]. The infection occurs through the stomata, leading to defoliation due to leaf curling and wilting from stomatal obstruction [18]. Leaf mold can cause wilting and floral organ abscission during flowering, reducing photosynthesis, nutrient uptake, and productivity [20]. The disease mostly affects tomatoes cultivated in greenhouses [17,19,22,23], but can occasionally affect field-grown tomatoes if conditions are favorable [19,24,25]. Containment is challenging once the disease has spread [23], causing 10–25% yield losses in normal years, and over 50% in high disease-pressure years [20]. *C. fulvum* has long been reported on tomato crops in Morocco [26].

The lack of resistant germplasm resources and variability in resistance has hindered the global screening for the best tomato leaf mold disease-resistance materials, slowing disease-resistance breeding development. Chemical pesticides remain the primary means to prevent and treat leaf mold disease [21]. However, resistant strains of *C. fulvum* to fungicides, such as azoxystrobin, a fungicide that binds to the cytochrome bc1 enzyme complex (complex III) at the Qo site to suppress the mitochondrial respiration of plant pathogens (Qo inhibitors, QoIs), have emerged [23], and pesticide use is restricted due to environmental concerns. Therefore, using microorganisms antagonistic to the pathogen could be a viable alternative [19]. Microbial-based biocontrol agents (BCAs) have shown promise in controlling plant diseases [27,28]. Thus, alternative methods for preventing and controlling *C. fulvum* infection that do not endanger the environment or human health, relying on BCAs, have been investigated [19,21,29,30,31]. 

Several bacterial and fungal species have been recognized as promising BCAs [32]. The effectiveness of BCAs, including *Trichoderma* spp., *Bacillus* spp., *Pantoea* spp. *Pseudomonas* spp., and *Streptomyces* spp., in preventing diseases caused by high humidity, such as tomato leaf mold, has been established [2,17,21,29,31,33]. A commercial biocontrol product based on an isolate of *Trichoderma harzianum* T39 successfully controlled *C. fulvum* and *Botrytis cinerea* on tomatoes, as well as *Sclerotinia sclerotiorum* and *B. cinerea* on cucumbers [29]. This isolate reduced tomato leaf mold disease by at least 77% [29]. In vitro and greenhouse conditions showed that *T. harzianum* was the most effective antagonistic fungus, reducing disease by 19.35% under greenhouse conditions [31]. *Bacillus subtilis* strain WXCDD105, isolated from the rhizosphere soil of a healthy tomato plant, effectively controlled *B. cinerea* and *C. fulvum,* also promoting tomato seedling growth [21]. The endophytic microbiome secretes diffusible antibiotic molecules, generates and releases volatile organic compounds (VOCs), and/or produces toxins to protect plants [34]. 

Given the potential for *C. fulvum* conidia to exceed 17,000 per square centimeter of an infected leaf during late vegetative stages [35], effective disease control measures are required. Chemical control techniques, primarily fungicides, are frequently used to manage fungal plant diseases [36]. Although azoxystrobin has a broad antifungal spectrum [37], its activity against *C. fulvum* is not well investigated. In vitro sensitivity experiments using mycelial homogenate grown on fungicide-added media were conducted due to the emergence of azoxystrobin-resistant *C. fulvum* isolates contributing to severe tomato leaf mold damage [23]. Additionally, the protective, curative, and eradicant activity of azoxystrobin against *C. fulvum* was determined in vivo.

The main objective of this study was to screen Moroccan bacterial and fungal isolates for their antagonistic activity against *C. fulvum*, and to evaluate the protective, curative, and eradicant effects of azoxystrobin on tomato plants infected with *C. fulvum*. Additionally, this study aimed to examine the impact of VOCs and diffusible secondary metabolites produced by these BCAs, to inhibit the mycelial growth of *C. fulvum*.

## 2. Results

### 2.1. Bacteria Effect on Fungal Mycelial Growth in an In Vitro Assay

The biocontrol efficacy of five bacterial isolates, *Bacillus amyloliquefaciens* SF14, *Alcaligenes faecalis* ACBC1, and *Pantoea agglomerans* (ACBC2, ACBP1, and ACBP2), was assessed in Petri dishes where they were co-cultured with *C. fulvum.* In vitro, all bacterial treatments significantly reduced the mycelial growth of *C. fulvum* compared to the untreated control (Table 1). The inhibition rate (%) of mycelial growth varied significantly across different incubation periods (5 and 12 days) and treatments (Figure S1). At the 5-day post-incubation (dpi) period, inhibition ranged from 34.8% to 54.4%, while at 12 dpi, inhibition ranged from 47.7% to 66.3%, with *P. agglomerans* P10c being the best (Table 1). Azoxystrobin was the only treatment achieving a nearly complete inhibition rate of 95.2%.

### 2.2. Culture Supernatant Effect on Fungal Mycelial Growth and Spore Germination

In vitro, all cell-free filtrates of the tested bacteria significantly reduced the mycelial growth of *C. fulvum* compared to the untreated control (Table 2). The inhibition rate (%) of the mycelial growth was influenced significantly by the incubation period (after 5 and 12 dpi) and treatments. At 5 dpi, three bacterial filtrates exhibited substantial inhibition rates exceeding 50%: *B. amyloliquefaciens* SF14 (52.4%), *A. faecalis* ACBC1 (51.2%), and *P. agglomerans* ACBP2 (50.0%). These values were similar to those of the reference bacteria *B. subtilis* Y1336 (55.2%) and *P. agglomerans* P10c (45.7%), which are products being sold commercially.

**Figure 1:**
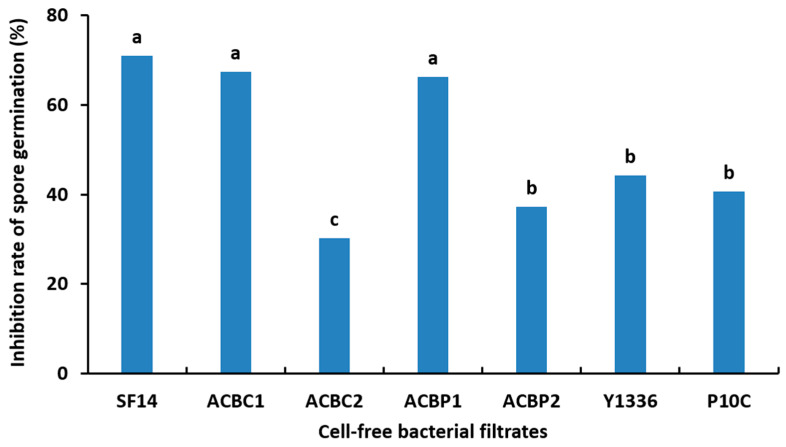
Spore germination inhibition rate (%) of *Cladosporium fulvum* in a total of 100 spores after incubation for 24 h, by cell-free filtrates of antagonistic bacteria at 100% concentration (*Bacillus amyloliquefaciens* SF14, *Alcaligenes faecalis* ACBC1, *Pantoea agglomerans* ACBC2, *P. agglomerans* ACBP1, *P. agglomerans* ACBP2, *Bacillus subtilis* Y1336, and *P. agglomerans* P10c). Data representing the average inhibition rate, with the same letter, are not significantly different according to the SNK test (*p* ≤ 0.05).

At the 12 dpi period, the inhibitory efficacy significantly improved, with *A. faecalis* ACBC1 achieving the highest inhibition rate at 57.44%, similar to the inhibition observed with the reference bacteria *B. subtilis* Y1336 (60.48%) and *P. agglomerans* P10c (58.68%). The remaining four bacterial filtrates demonstrated inhibition rates ranging from 38 to 53% (Table 2). The cell-free filtrates of the tested bacteria at a concentration of 2% effectively suppressed the spore germination of *C. fulvum*, as illustrated in Figure 1. *B. amyloliquefaciens* SF14 demonstrated a notable inhibition rate of 70.9%. The cell-free filtrates of *P. agglomerans* ACBP1 and *A. faecalis* ACBC1 exhibited inhibition rates between 60% and 70% for spore germination, along with the reference commercial bacteria *B. subtilis* Y1336 (44.18%) and *P. agglomerans* P10c (40.69%), which achieved inhibition rates below the average of 50%.

### 2.3. Bacteria-Based VOCs Effect on the Fungal Mycelial Growth

In vitro, all bacterial VOCs significantly reduced the mycelial growth of *C. fulvum* compared to the untreated control (Table 3). The inhibition rate (%) of the mycelial growth was significantly influenced by the periods of incubation (after 5 and 12 dpi) and treatments. At 5 dpi, *B. amyloliquefaciens* SF14 achieved an inhibition rate of 57.4%, outperforming the reference bacteria *B. subtilis* Y1336 (54.4%) and *P. agglomerans* P10c (54.4%). Other isolates showed inhibition rates between 30% and 55%. By 12 dpi, *B. amyloliquefaciens* SF14 demonstrated the highest inhibition rate at 67.4%, while *P. agglomerans* ACBP2 showed the lowest inhibition rate at 44.2% (Table 3).

### 2.4. In Vitro Effects of Antagonistic Fungi on the Fungal Mycelial Growth

In vitro, all biological treatments with the three *Tricoderma* spp. isolates led to complete inhibition of the mycelial growth of *C. fulvum* at both 5 dpi and 12 dpi (Table 4; Figure S2). These isolates even outperformed the effectiveness of the commercial chemical fungicide azoxystrobin (95.9%) (Table 4).

### 2.5. In Vitro Effects of Chemical Fungicide on the Pathogen Mycelial Growth

In vitro, all chemical treatments significantly reduced the mycelial growth of *C. fulvum* in comparison with the untreated control (Table 5). All three tested doses (5, 10, and 20 µg a.i.mL^−1^) of the fungicide exhibited significant differences when compared to the control. At the 5 dpi period, all treatments demonstrated inhibition rates exceeding 90%, and these values are similar to the inhibition rates achieved by the reference fungicide (Table 5). At 12 dpi, there was no further improvement in the inhibition rate in any of the treatments (Table 5). It should be noted that there is no significant difference between day 5 (91.2%) and day 12 (91.7%).

### 2.6. In Plant Bioassays

#### 2.6.1. Effect of Microorganisms in Greenhouse Conditions

**Figure 2:**
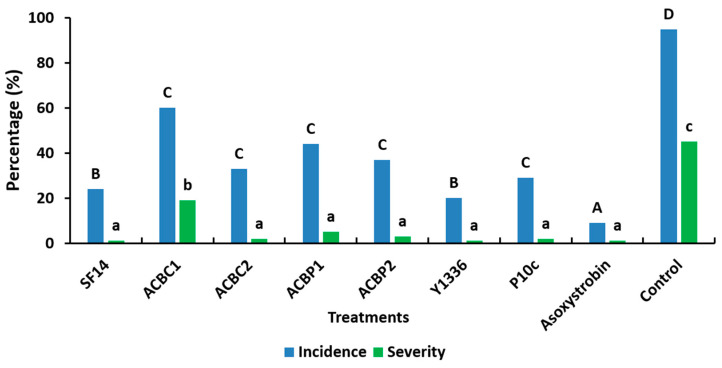
Observation of disease severity and incidence on the leaf of tomato plants treated with the seven tested bacterial (*Bacillus amyloliquefaciens* SF14, *Alcaligenes faecalis* ACBC1, *Pantoea agglomerans* ACBC2, *P. agglomerans* ACBP1, *P. agglomerans* ACBP2, *Bacillus subtilis* Y1336, and *P. agglomerans* P10c) suspensions (10^8^ CFU/mL) and inoculated with *Cladosporium fulvum*, after 30 days of incubation at 25 °C within greenhouse conditions. Control: positive control (pathogen only; *C. fulvum*). Asoxystrobin: plants treated with fungicide. Bar charts represent the mean value of disease severity of two trials over time with four replicates. Values of plant incidence and severity with the same letter (uppercase for incidence: A, B, etc.; lowercase for severity: a, b, etc.) were not significantly different according to the SNK test (*p* ≤ 0.05).

The five bacterial isolates were evaluated in the greenhouse to assess their inhibitory effects and abilities to control leaf mold disease. All the tested bacteria significantly affected the disease incidence and severity in comparison with the untreated control (pathogen only) (Figure 2). In the control plants, the incidence was 95%, whereas it ranged between 20% and 60% in the treated plants. The disease incidence and severity were influenced significantly by the treatments. A significant difference between all treatments and the control was indicated. Moreover, these outcomes manifested as varying incidences and severities contingent on the bacterial isolates (Figure 2). At the 30 dpi period, the bacteria exhibited an average severity of 4.8%. The range extended from a minimum of 0.19% (*B. subtilis* Y1336) to a maximum of 19.5% (*A. faecalis* ACBC1), whereas the control exhibited a severity of 42.4%. The application of antagonistic sprays in the greenhouse significantly mitigated tomato leaf mold incidence, with disease reduction spanning approximately 36.8% to 78.9%, contingent on the bacterial isolates. Five bacterial isolates demonstrated a high reduction in severity exceeding 90% in comparison to the control: *B. subtilis* Y1336 (99.5%), *B. amyloliquefaciens* SF14 (98.2%), *P. agglomerans* P10c (95.3%), *P. agglomerans* ACBC2 (95.3%), and ACBP2 (90.9%), akin to the azoxystrobin fungicide (99.8%). One bacterial isolate (*P. agglomerans* ACBP1) achieved a severity reduction of 87%, categorizing it between 60% and 90% compared to the azoxystrobin fungicide (99.8%). Another bacterial isolate (*A. faecalis* ACBC1) exhibited a lesser severity reduction (below 60%) of approximately 54%.

The three *Trichoderma* spp. isolates were evaluated in the greenhouse to assess their inhibitory effects and abilities to control leaf mold disease. All the tested isolates significantly affected the disease incidence and severity in comparison with the untreated control (Table 6). The disease symptoms on day 30 were obvious (Figure S3). In the control plants, the incidence was approximately 94%, whereas it ranged between 3% and 10% in the treated plants. At 30 dpi, the *Trichoderma* isolates reduced severity by 98.2 to 99.7%, which is higher than the 77.9% reduction provided by azoxystrobin fungicide (Table 6).

#### 2.6.2. Effect of Commercial Fungicide

Three doses (5, 10, and 20 µg a.i.mL^−1^) of the fungicide azoxystrobin were evaluated in the greenhouse and were found to significantly reduce disease occurrence. The disease occurrence was influenced significantly by the result of the harvest time (before the inoculation of the plant (IOP) with *C. fulvum* (protective activity), following IOP with *C. fulvum* (curative activity), and post-symptom activity) and treatments. A significant difference between all treatments and the control was observed.

##### Protective Activity

Both azoxystrobin and chlorothalonil chemical fungicides provided significantly (*p* < 0.05) less disease severity (Table 7). Azoxystrobin at a dose of 20 μg a.i.mL^−1^ provided the greatest control overall and even when applied 96 h before IOP, it provided a >90% reduction in lesions (Table 7). Azoxystrobin D10 and chlorothalonil D100 led to a >90% reduction in severity when applied 48 h before IOP, but azoxystrobin D5 did not attain a >90% reduction even when applied 24 h before IOP.

##### Curative Activity

After pathogen inoculation, applying azoxystrobin D20 within 24 and 48 h after IOP displayed a remarkable control efficacy exceeding 90%. However, using the same dose 96 h after IOP resulted in a control efficacy of only 66.5% (Table 8). Employing azoxystrobin D10 either 24 or 96 h after IOP led to disease-severity levels similar to those observed in plants treated with azoxystrobin D20. Notably, a reduction in disease severity was observed with an application 48 h after IOP. In contrast, azoxystrobin D5 demonstrated reduced effectiveness compared to the D20, regardless of the application time. Chlorothalonil, serving as a protective fungicide, showcased effective disease control solely when applied 24 h after IOP. Conversely, applications of chlorothalonil 48 or 96 h after IOP resulted in control levels of 74.3% and 48%, respectively (Table 8).

##### Post-Symptom Activity

The production of spores on plants subjected to fungicide treatment after symptom emergence exhibited a significant reduction compared to the untreated control plants (Table 9). Azoxystrobin D20 displayed the most pronounced anti-sporulation activity with azoxystrobin D5 the lowest and azoxystrobin D10 and chlorothalonil D100 intermediate (Table 9).

### 2.7. Efficacy Test of Bacterial Strain Candidates for Commercialization as a Biological Product

**Figure 3:**
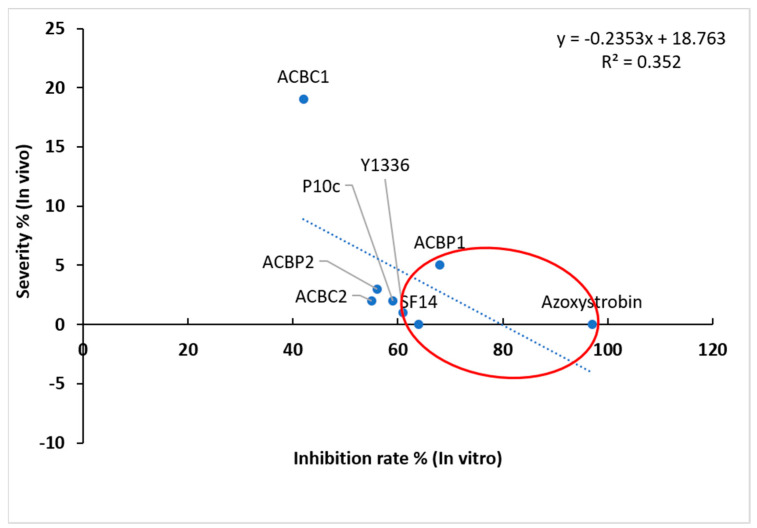
Linear regression of severity % (in vivo) and inhibition rate % (in vitro) of the seven tested bacteria (*Bacillus amyloliquefaciens* SF14, *Alcaligenes faecalis* ACBC1, *Pantoea agglomerans* ACBC2, *P. agglomerans* ACBP1, *P. agglomerans* ACBP2, *Bacillus subtilis* Y1336, and *P. agglomerans* P10c) and the commercial fungicide against *Cladosporium fulvum*.

The conducted in vitro and in vivo tests have highlighted the robust antagonist activity of various bacterial species against *C. fulvum.* From these, bacterial strains exhibiting the highest potential were chosen for further experiments, involving the inoculation of *C. fulvum* on tomato leaves under conditions akin to large-scale production in greenhouses. The selection of these bacteria was based on the following criteria: resulted in minimum severity of mold disease after application in vivo, achieving the highest inhibition rates in vitro, and exhibiting inhibition levels that align with the consistent performance of the fungicide across various results. The two selected bacterial strains were *B. amyloliquefaciens* SF14 and *P. agglomerans* ACBP1 (Figure 3).

**Figure 4:**
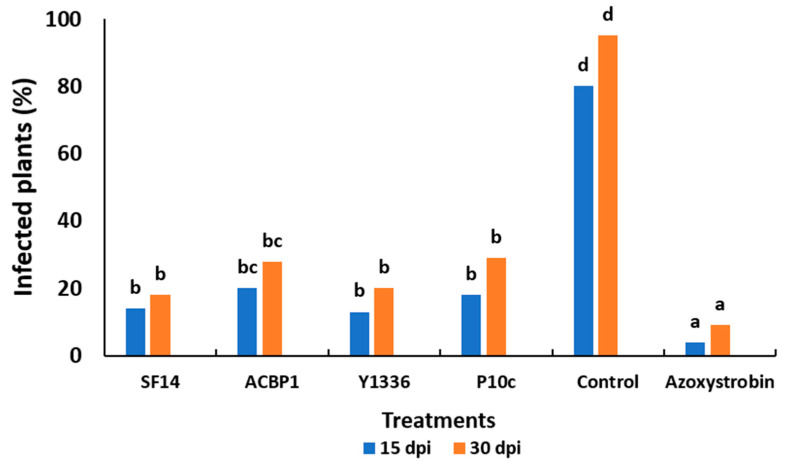
The average percentage of infected tomato plants (%) for different treatments (*Bacillus amyloliquefaciens* SF14, *Alcaligenes faecalis* ACBC1, *Pantoea agglomerans* ACBC2, *P. agglomerans* ACBP1, *P. agglomerans* ACBP2, *Bacillus subtilis* Y1336, *P. agglomerans* P10c, and the commercial fungicide against *C. fulvum*) recorded after 15 and 30 days of artificial inoculation incubated at 25 °C and 85% HR. Treatments with the same letter are not significantly different according to the SNK test (*p* ≤ 0.05).

The progression of leaf mold disease induced by *C. fulvum* was influenced significantly by the periods of incubation (after 15 and 30 days) and treatments (comprising the two selected antagonistic bacteria, the two reference bacteria, and the chemical fungicide). As shown by the SNK test, all treatments were significantly different from the untreated control (Figure 4). The prevalence of infected plants progressively rose with the duration of incubation across all treatment groups. At the end of 30 dpi, the incidence of infected fruits ranged from 9% to 27% in all treatment categories, whereas the control group exhibited a significantly higher infection rate of 95%. Among the selected strains, *B. amyloliquefaciens* SF14 displayed a robust protective effect against *C. fulvum*, resulting in an infection rate of 18% among plants. *P. agglomerans* ACBP1 demonstrated a level of protection (27%) comparable to that of the reference bacteria *B. subtilis* Y1336 (20%) and *P. agglomerans* P10c (29.5%). Nonetheless, both strains exhibited lower efficacy compared to the azoxystrobin fungicide (9%).

### 2.8. In Vivo Phytochemical Analysis by the FTIR Technique

Using FTIR spectroscopy, the active component’s functional groups were identified based on peak values within the infrared radiation spectrum. The outcomes of the most dominant FTIR peak values and associated functional groups are displayed in Table 10. The infrared (IR) spectra of tomato leaves exhibited multiple absorption peaks, indicating the intricate nature of the examined biomass. To discern disparities between treatment peaks, the results depicted in Table 10 unveiled distinctive absorbance patterns, characterized by diverse peak shapes suggesting the potential presence of phenolic, carboxylic, hydroxyl, and carbonyl groups. The FTIR spectra have corroborated shifts in functional groups and surface attributes of the absorbent, contrasting them with the control leaf spectrum. Bands linked to various chemical groups within the fingerprint region (1800–800 cm^−1^), encompassing pectin (C=O at 1740 cm^−1^) and hemicellulose (1248 cm^−1^), are pinpointed and enumerated in Table 11. The 1655 cm^−1^ band (Amide I) corresponded to C=O and N-H vibrations, while the 1546 cm^−1^ band (Amide II) was linked to N-H and C-N vibrations. The C=C stretching of the aromatic ring within lignin was noted at both 1610–1590 cm^−1^ and 1515–1505 cm^−1^. For cellulose, bands manifested at 1372, 1161, and 1060 cm^−1^, with the 995 cm^−1^ band associated with C-C ring vibration.

Evident from the spectral absorptions in the leaves, the treatments administered to tomato plants through pulverization led to biochemical modifications in the cellular composition of the treated plants, contrasting with the controls. Substantial distinctions surfaced between the treated and untreated tomato plants. Considering the spectral absorptions in the FTIR spectra, the pulverization of various treatments induced biochemical changes in the leaves distinct from those of the control (Table 11).

In the plants subjected to all treatments, the integrated area’s content for absorption bands linked to C=C stretching of the aromatic ring vibration in lignin (1615–1590 and 1463), hemicellulose (1210 cm^−1^), cellulose (1066 cm^−1^), and protein amide I (1535 cm^−1^) persisted post-pulverization. Changes resulting from treatment pulverization were noted at wavenumbers 1737, 1668, 1606, and 1426 cm^−1^, conceivably attributed to pectin, proteins, and lignin, respectively. Notably, all *Trichoderma* spp. isolates induced a marked reduction in the integrated area of pectin and cellulose bands, concomitant with an increase detected in proteins and lignin, which could be attributed to the presence of the fungus. 

## 3. Discussion

The prevalent use of agrochemicals in modern agricultural production systems raises concerns about the sustainability of future food supplies. For some time now, a move toward cutting-edge environmentally friendly alternatives to chemical pesticides has been necessary due to the clear negative impacts on the environment and unforeseen impacts on human and animal health [28,38,39]. BCAs have emerged as an important alternative to synthetic fungicides in controlling *C. fulvum*-caused tomato leaf mold [19,21,29,30,31]. Various isolates of the genera *Bacillus, Pantoea*, and *Trichoderma* are known for their ability to protect plants against many pathogens and have been identified as potential BCAs [33,34,40,41,42,43,44,45,46].

*Bacillus* species are among the most widely used microbial constituents due to their highly effective mechanisms of action against plant pathogens, as well as additional plant-beneficial properties and suitability for microbial biopesticide formulations [28,47]. Potential BCAs are screened based on their antagonistic activity. In this context, the purpose of the current study was to assess the antagonistic activity of five Moroccan bacterial isolates and three fungal isolates from the collection of the laboratory phytopathology (ENA-MEKNES), which had previously shown promising antagonistic effects against various pathogens [41,42,44,48], in controlling *C. fulvum* on tomatoes. These isolates and their secondary metabolites (cell-free bacterial filtrates and VOCs) were subjected to both in vitro and in vivo studies. The antifungal efficacy of these biological treatments was compared to those of biological and chemical reference products. Previous research has documented the impact of cell-free supernatants from bacterial cultures and bacteria-emitted VOCs on the mycelial growth and spore germination of phytopathogenic fungi [34,49].

Overall, compared to the controls, the presence of bacterial isolates and their secondary products significantly decreased mycelial development. The results indicated that the inhibition rates varied between direct dual bioassay (direct effect), aseptic bacterial filtrates, and bacteria-based VOCs (long-distance effect). This variation may be explained by the fact that bacterial isolates utilize a variety of mechanisms to exert their antagonistic activities, depending on the nature of the pathogen [42].

Our findings showed that bacterial isolates exhibited significant in vitro antagonistic capacity against *C. fulvum* mycelial growth, ranging from 34 to 51% at 5 dpi, increasing to 47–63% at 12 dpi, which is consistent with previous results that showed growth inhibition rates increased with time [43]. The highest inhibition rates were observed with *P. agglomerans* isolates ranging from 60.4 to 62.2%, close to the two reference bacteria (65.0, 66.3%) currently being sold commercially, but lower than the chemical fungicide (95.9%). *B. amyloliquefaciens* SF14 at 57.21% and *A. faecalis* ACBC1 at 47.7% were lower. Previous studies have also reported the antagonistic effects of some of these bacterial isolates against plant diseases [42,43,44]. In particular, *B. amyloliquefaciens* SF14 and *P. agglomerans* ACBC2 were notably effective against *Pythium schmitthenneri* growth, showing inhibition rates of 81.8% and 80.6%, respectively, at a 6 dpi period [44]. Our results are consistent with findings that *Bacillus* spp. and *Pantoea* spp. tend to exhibit strong in vitro antifungal activity compared to other bacterial species. Strong antifungal activity was observed against *C. fulvum* by bacterial isolates assumed to be *B. subtilis*. Additionally, several *Pantoea* species displayed inhibitory zones when exposed to *C. fulvum* [33]. The inhibitory impact of the tested bacterial isolates could be attributed to the fact that *Pantoea* and *Bacillus* species are known to produce and secrete numerous lipopeptides to combat fungal pathogens [50,51]. To test this hypothesis, we investigated the antifungal activity of cell-free supernatants and VOCs against *C. fulvum.*

The aseptic filtrates from bacterial isolates showed a significant inhibitory effect on the growth of the pathogen causing tomato leaf mold disease. The hyphae of *C. fulvum* grew more slowly in Petri dishes containing bacterial isolates compared to the untreated control dishes. The inhibition rate of *A. faecalis* ACBC1 aseptic filtrates on the growth of *C. fulvum* was 57.4% at 12 dpi, similar to that of the commercial bacteria *B. subtilis* Y1336 (60.5%) and *P. agglomerans* P10c (58.7%). The other aseptic filtrates were less effective. Wang et al. [21] also reported the inhibition of in vitro mycelial growth of *C. fulvum* in the presence of aseptic filtrates from the *B. subtilis* WXCDD105 isolate, suggesting the presence of antifungal compounds. Furthermore, cell-free bacterial filtrates demonstrated inhibitory effects on the spore germination of *C. fulvum* under in vitro conditions. *B. amyloliquefaciens* SF14 showed the highest inhibition rate at 70.9%, significantly outperforming the commercial bacteria *B. subtilis* Y1336 (44.2%) and *P. agglomerans* P10c (40.7%).

VOCs, among other bioactive metabolites, have been extensively studied for their biocontrol applications, particularly due to their ability to act over long distances without direct contact with plants or pathogens [28]. Consequently, one of the objectives of this study is to provide an updated overview of bacteria-based VOCs, especially regarding their antifungal effects, which are critical for their potential use in sustainable agriculture. 

The bacteria-based VOCs demonstrated a significant inhibitory effect on the growth of the pathogen causing tomato leaf mold disease. At the 12 dpi period, *B. amyloliquefaciens* SF14 showed the highest inhibition rate on the growth of *C. fulvum* at 67.5%, surpassing the reference bacteria *B. subtilis* Y1336 (65.0%) and *P. agglomerans* P10c (62.6%). The differences in inhibition rates could be due to the varied interactions between each bacterium and the fungus, resulting in the production and release of different VOCs [34]. 

Following the in vitro experiments, our bioassay results on tomato plants highlighted that *B. amyloliquefaciens* SF14 exhibited the lowest disease severity of leaf mold of 0.8% at 30 dpi, comparable to the commercial bacteria *B. subtilis* Y1336 (0.2%) and *P. agglomerans* P10c (2.0%), and the chemical fungicide (0.1%). These results are consistent with those of Wang et al. [21], who found that the antagonistic effect of *B. subtilis* WXCDD105 against *C. fulvum* was highly effective in vivo and better than that of chemical pesticides.

As with bacterial isolates, our findings showed that fungal isolates had a significant in vitro antagonistic capacity against *C. fulvum* mycelial growth, achieving a 100% inhibition rate within just 5 dpi, comparable to the action of the chemical fungicide. In subsequent bioassays on tomato plants, *Trichoderma* spp. BT3 exhibited the lowest disease severity of tomato leaf mold of 0.1% during the 30 dpi period, comparable to the chemical fungicide, which showed a severity level of 7%. These findings align with the known effectiveness of *Trichoderma* spp. as promising BCAs that are mass-produced, formulated, and commercially available for agricultural use [32]. Additionally, previous studies have shown the effectiveness of *Trichoderma* isolates, such as *T. harzianum* T39, in managing *C. fulvum* in vivo. This BCA effectively controlled *C. fulvum* on tomato plants grown commercially in greenhouses [29]. 

The effects of the fungicide azoxystrobin on *C. fulvum* mycelial growth, as well as its protective, curative, and eradicant activity against *C. fulvum*, were evaluated under both in vitro and in vivo conditions. Azoxystrobin at 20 µg a.i.mL^−1^ inhibited *C. fulvum* mycelial growth by 97.2% after 5 dpi and by 97.1% after 12 dpi. These results are comparable to, or even superior to, those of chlorothalonil at 100 µg a.i.mL^−1^, which inhibited growth by 98.3% and 95.6% at 5 and 12 dpi, respectively. The in vitro test results demonstrate that the Moroccan isolate of *C. fulvum* is sensitive to azoxystrobin, and that this fungicide can impede mycelial growth even at low concentrations, with inhibition rates increasing with higher fungicide concentrations. This finding differs from results indicating that *C. fulvum* isolates from Japan require a minimum inhibitory concentration of 31–500 µg a.i.mL^−1^ for azoxystrobin sensitivity, and 8,000–32,000 µg a.i.mL^−1^ for resistance [23]. Azoxystrobin provided excellent control of tomato leaf mold disease, especially in protective treatments. It interferes with the tomato leaf surface by preventing pathogen spore production, thereby limiting the early spread of the disease. The sporulation inhibition rate of *C. fulvum* increases with higher concentrations of azoxystrobin. Remarkably, using azoxystrobin at only 10 µg a.i.mL^−1^ produces results comparable to those obtained with chlorothalonil at 100 µg a.i.mL^−1^. These outcomes are similar to those obtained with trifloxystrobin, another strobilurin fungicide, which also provides excellent control of tomato leaf mold disease when applied as a preventive measure [35]. Our experiments demonstrated that while azoxystrobin had a significant curative effect, its efficacy was inferior to that of protective treatments, particularly when applications were made 96 h after IOP. Application of 20 µg a.i.mL^−1^ of azoxystrobin 48 h after IOP resulted in a control efficacy of 94.6%, whereas lower doses of 5 and 10 µg a.i.mL^−1^ achieved control efficacies of 60.5% and 76.4%, respectively. These are comparable to the standard fungicide chlorothalonil at 100 µg a.i.mL^−1^ (74.3%). The modest curative effect of strobilurin fungicides, including azoxystrobin, is likely due to their ability to prevent fungal growth within leaf tissues [35]. These findings align with previous reports indicating that strobilurin fungicides have modest curative action against various diseases [52], including *C. fulvum* [35]. The high anti-sporulant activity of azoxystrobin against *C. fulvum* is particularly important for managing this polycyclic disease, as reducing spore production can help inhibit further disease development. This high anti-sporulant activity has also been observed with trifloxystrobin [35]. To the best of our knowledge, this is the first study to evaluate the efficacy of azoxystrobin against tomato leaf mold disease.

The FTIR spectrum results obtained in this study demonstrated absorption signals for multiple wavenumber ranges, which were identified as corresponding to pectin, proteins, lignin, hemicellulose, and cellulose [53]. The obtained spectrum constitutes the fingerprint of a treatment, highlighting characteristic peaks (or bands) of various chemical bonds and organic groups present in the studied leaf biomass.

This study underscored the impact of different treatments (bacteria, fungi, and fungicides) on the chemical composition of the leaves. The FTIR spectra confirmed changes in functional groups, particularly within the broad range of 800 to 1800 cm^−1^. These changes can be attributed to the complexation of bacterial and fungal component ions with the ionized O-H groups of hydroxyl groups and bonded O-H bands of carboxylic acids in the inter- and intramolecular hydrogen bonding of polymeric compounds, such as alcohols, phenols, and carboxylic acids, as found in pectin, cellulose, and lignin [54].

Alterations in the band between 2940 and 2920 cm^−1^ and at 1424.2 cm^−1^ indicate an ion-exchange process involving the protons of asymmetric C-H and the symmetric stretching vibration of CH₂, respectively, of aliphatic acids present in the biomass and the fungal treatment ions. Changes in peaks observed at 1700 and 1780 cm^−1^, representing the stretching vibration of C=O bonds due to non-ionic carboxyl groups (-COOH, -COOCH₃), may be attributed to hydrogen-bonding carboxylic acids or their esters and the treatment ions.

Generally, the treatments (bacteria, fungicide) did not show a significant difference in cell composition. However, *Trichoderma* spp. isolates suppressed the detection of some cell components, such as pectin, lignin, and cellulose. This work is the first to use spectroscopy to determine the differences in biochemical composition in the tissue of tomato leaves. The subtle changes identified in chemical composition following treatment application may serve as a reference to evaluate whether these treatments negatively impact tomato development and production.

## 4. Materials and Methods

### 4.1. Biological Material and Culture Conditions

#### 4.1.1. Fungal Pathogen

*C. fulvum* was obtained from tomato plants grown in a greenhouse in the Souss-Massa region of Morocco, which displayed moldy lesions on their leaves. The affected plants were removed from the greenhouse, and the leaves with lesions were carefully cut into small pieces and subjected to a series of treatments. Firstly, they were disinfected by soaking in a 5% sodium hypochlorite solution for 2 min, followed by rinsing in ethanol and sterile distilled water (SDW). Subsequently, the treated leaf pieces were air-dried on sterilized Whatman filter paper within a laminar flow cabinet. Once fully dried, these leaf segments were placed onto Petri dishes containing Potato Dextrose Agar (PDA). The dishes were then kept in the dark at a temperature of 25 °C for incubation.

Another method of isolation involved extracting leaf segments with spore-producing lesions using a sterile cork borer (5 mm in diameter). These segments were placed in Eppendorf tubes containing 3 mL of SDW. Through vortexing, conidia were detached from the lesions on the leaf segments. After removing the leaf segments, a 2 µL sample of the conidial suspension was deposited onto PDA in Petri dishes, which were then incubated at 25 °C for 21 days. Following incubation, individual colonies were selected, and the pathogenic fungus was isolated and purified.

For subsequent experiments, the fungal colonies used were obtained from cultures that were 10 days old and had been incubated at 25 °C. The suspension of spores from *C. fulvum* was obtained by gently scraping a pure colony aged 10 to 15 days using a sterile, pointed needle, after introducing 15 mL of SDW containing 0.05% Tween 20. The resultant spore suspension was then filtered through four layers of cheesecloth to eliminate any debris or mycelial fragments, leaving only the spores in the suspension. The concentration of the suspension was then adjusted to the desired final concentration (1 × 10^6^ conidia/mL) using a Malassez cell.

#### 4.1.2. Antagonistic Fungi

This study utilized three strains of *Trichoderma* spp. obtained from the soil surrounding olive (isolate OT1), apple (isolate AT2), and blueberry plants (isolate BT3) (Figure S4). To ensure a consistent supply, these strains were cultivated on PDA medium under dark conditions at 25 °C for a period of 10 to 15 days.

#### 4.1.3. Antagonistic Bacteria

The bacterial isolates used in this study, namely *B. amyloliquefaciens* SF14, *A. faecalis* ACBC1, and *P. agglomerans* (ACBC2, ACBP1, and ACBP2), were obtained from soil and rosaceous blossoms across various regions of Morocco. These isolates were previously identified, sequenced, and evaluated for their potential as BCAs [48]. To ensure their long-term viability, the bacterial isolates were stored at −20 °C in vials containing 30% glycerol within the Phytopathology Unit Laboratory at ENA-Meknes. Before conducting experiments, the bacterial isolates were subcultured on a PDA medium under dark conditions at 25 °C for 48 h. Their concentration was adjusted to 1 × 10^8^ CFU/mL using a spectrophotometer at a wavelength of 620 nm. Additionally, their antagonistic effects were compared with those of two reference biofungicides used as controls: *Bacillus subtilis* Y1336 (Biopack) and *Pantoea agglomerans* P10c (PomaVita).

### 4.2. Tested Chemical Fungicide

The chemical fungicide, azoxystrobin (Priori^®^Top, Syngenta S.A, Basel, Switzerland), was also tested. In the chemical control tests, it was utilized at varying concentrations: 1, 5, 10, 20, and 50 µg a.i./mL. For the reference fungicide in the chemical experiments, Daconil 720 SC, containing chlorothalonil from the organochlorine family, was selected. Chlorothalonil (Daconil 720 SC, Syngenta S.A) is a fungicide known for its protective effects against *C. fulvum* and is commonly employed as a standard fungicide treatment at a dosage of 100 µg a.i./mL. In the biological control trials, azoxystrobin was applied at a concentration of 20 µg a.i./mL.

### 4.3. In Vitro Effects of Biological and Chemical Treatments on C. fulvum Mycelial Growth

#### 4.3.1. Effect of Antagonistic Bacteria

To assess the impact of the five chosen bacterial isolates on restraining *C. fulvum* growth, both 24 h old bacterial cultures and 7 to 10-day-old mycelial cultures were employed. Using an inoculation loop, a bacterial suspension, prepared as described by Legrifi et al. [44], was transferred onto a PDA medium. On the PDA surface, four parallel lines of bacterial streaks (each around 3 to 4 cm in length) were applied. Following this, a mycelial disk measuring 5 mm was placed inverted at the center of the PDA medium. For comparison, a negative control was set up using PDA alone, containing solely the mycelial disk of *C. fulvum* without any bacterial streaks. Azoxystrobin was utilized as a positive control.

The experiment was structured with nine distinct treatments for two incubation times (5 and 12 days post-incubation (dpi) at 25 °C). For each incubation time, each treatment was replicated four times, following a completely randomized block design. The test was repeated twice over time.

#### 4.3.2. Effect of Bacterial Cell-Free Filtrates

To explore the indirect effect of the antagonistic bacteria on mycelial growth, bacterial cell-free filtrates containing only bacterial metabolites were assessed by adding the filtrate to the center of the PDA media, and the mycelial disk was placed as described for the antagonistic bacteria. The preparation of bacterial filtrates was conducted following the method outlined by Li et al. [49] with modifications. The five tested bacteria were initially cultured on a solid Nutrient Agar (NA) medium through streaking and subsequently incubated at 25 °C for 48 h. After disinfecting with 70% ethanol and sterilizing under UV radiation in a laminar flow hood, the bacterial colonies were collected using a sterile loop and transferred to tubes containing liquid NA medium. These tubes were then placed on a shaker and grown at 28 °C for 3 days (130 rpm). To obtain the filtrate solution, the culture was centrifuged at 5000 rpm for 28 min, allowing the bacterial pellet to settle at the tube’s bottom and the supernatant to remain on top. The bacterial supernatant was carefully collected and subsequently filtered through a 0.22 µm syringe filter. The obtained filtrates were stored at −20 °C until use. For comparison, fresh cells of the reference bacteria *B. subtilis* Y1336 and *P. agglomerans* P10c were prepared through serial dilution using commercial products, and they were applied following the same protocol. Negative controls consisted of Petri dishes with only the pathogen, while positive controls featured the chemical fungicide azoxystrobin.

The experiment was structured with eight distinct treatments for two incubation times (5 and 12 dpi). For each incubation time, each treatment was replicated four times, following a completely randomized block design. The test was repeated twice over time.

#### 4.3.3. Effect of Volatile Organic Compounds

On Luria–Bertani (LB) medium, the antagonistic bacteria were cultivated in three streaks and incubated at 28 °C. After 24 h, the Petri plate lid of the bacterial cultures was exchanged with the base of another Petri plate containing a 5 mm fungal disk from a 10-day-old mycelial culture on PDA. Parafilm was used to seal the bases of the two Petri dishes. A control was prepared similarly but without the bacterial culture. The experiment was structured with eight distinct treatments for two incubation times (5 and 12 dpi at 25 °C). For each incubation time, each treatment was replicated four times, following a completely randomized block design. The test was repeated twice over time.

#### 4.3.4. Effect of Antagonistic Fungi

The confrontation test was conducted using 10-day-old cultures of the three *Trichoderma* spp. isolates and a 7 to 10-day-old *C. fulvum* mycelial culture. In each Petri dish, a mycelial disk (5 mm) from the *Trichoderma* spp. isolate and another mycelial disk (5 mm) from the *C. fulvum* pure culture were placed, maintaining a distance of 3 to 4 cm between them. For control purposes, separate Petri dishes were prepared with only the mycelial disk and no treatment, one with the *Trichoderma* spp. isolates and the other with *C. fulvum.* The experiment was structured with five distinct treatments for two incubation times (5 and 12 dpi at 25 °C). For each incubation time, each treatment was replicated four times, following a completely randomized block design. The test was repeated twice over time.

#### 4.3.5. Effect of Chemical Fungicide

Before pouring PDA into the Petri dishes, three varying concentrations of azoxystrobin (5, 10, and 20 ppm) were introduced. The PDA with amendments was centrally inoculated with a 5 mm disk of a 7 to 10-day-old *C. fulvum* colony. The plates were then incubated in darkness at 25 °C. A Petri dish containing only PDA inoculated with the pathogen, without any fungicidal amendments, served as the negative control. As a positive control, Daconil 720 SC, a fungicide known for its protective effect against *C. fulvum*, was included. It was added to the PDA before the pathogen’s inoculation at a concentration of 100 µg a.i.mL^−1^. The experiment was structured with five distinct treatments for two incubation times (5 and 12 dpi at 25 °C). For each incubation time, each treatment was replicated four times, following a completely randomized block design. The test was repeated twice over time.

#### 4.3.6. Assessing *C. fulvum* Mycelial Growth 

For all the in vitro assays, the diameter (mm) of the mycelial growth was then recorded at 5 and 12 dpi periods [45]. The inhibition rate (IR) was calculated based on the following formula:

IR (%) = (D_C_ − D_T_)/(D_C_ − D_0_) × 100

where

D_C_: colony diameter (mm) of *C. fulvum* in the control (culture medium without BCA).
D_T_: colony diameter (mm) of *C. fulvum* in the treatment (PDA medium with BCA).
D_0_: diameter of the mycelial disk (5 mm).

### 4.4. Effect of Bacterial Cell-Free Filtrates on Spore Germination

To assess spore germination inhibition, a combined mixture consisting of 1 mL of *C. fulvum* spore suspension (1 × 10^6^ spores per ml) and 1 mL of bacteria cell-free filtrate suspension was introduced into 50 mL Potato Dextrose Broth (PDB) medium. The control was prepared in the same manner but without the bacterial filtrate. This composite solution was then subjected to incubation at 26 °C with agitation at 75 rpm for 24 h. Subsequently, the germinated spores were quantified using a light microscope. The inhibition of spore germination was determined using the following formula:

Ig = (G_C_ − G_T_)/G_C_ × 100

where

G_C_: count of germinated spores in the negative control (without any treatment).
G_T_: number of germinated spores in the treatment (with bacterial filtrate).

### 4.5. Greenhouse Bioassays

#### 4.5.1. Plant Material

In vivo experiments were conducted using a highly susceptible tomato cultivar (Campbell 33) for tomato leaf mold disease. Tomato seedlings were cultivated in a greenhouse maintained at temperatures between 18 and 26 °C. After three weeks, the seedlings were transplanted into experimental pots measuring 7.5 cm in diameter and 9 cm in depth. These pots were filled with a mixture of peat and perlite in a 10:1 ratio. The soil underwent three sterilization cycles at 121 °C for 30 min each, with 24 h intervals between cycles. Each pot was dedicated to a single plant. Throughout the experiments, the plants were watered twice daily (early morning and late evening) and fertilized once per week with a 1% N:P:K (20:20:20) solution.

#### 4.5.2. Treatment with Antagonistic Bacteria/Fungi or Chemical Fungicide and Plant Inoculation

Regardless of the specific trial, inoculation of the plants (IOP) occurred seven weeks after seeding, at a growth stage characterized by the presence of 7–8 fully expanded leaves. Azoxystrobin was used as a positive control, and water served as the negative control for plants inoculated with *C. fulvum.* These controls were applied simultaneously with the antagonistic bacteria and fungi treatments. The transplantation process involved spacing the plants 50 cm apart, with 80 cm between each block. To prevent the drift of the spray solution across experimental plots, one tomato plant between these plots was intentionally left unsprayed. Results for both antagonistic bacterial and fungal treatments were assessed at 15 and 30 dpi. Results from the fungicide experiment were evaluated at three time points: before IOP, immediately after IOP, and post-IOP.

##### Antagonistic Bacteria 

The experiment was structured with nine distinct treatments, each replicated four times, following a completely randomized block design. Within each experimental block, seventeen plants were present, spanning a length of eight meters. Sprays were applied both before any disease symptoms were visible on the plants (prevention strategy) and after the initial appearance of symptoms (curative strategy). The initial spray application was executed upon the full growth of the eighth leaf, with subsequent applications repeated at 10-day intervals until pathogen inoculation (for prevention). Tomato plants underwent a three-week treatment with a liquid suspension of each bacterial strain (1 × 10^8^ CFU/mL), applied once a week through spray inoculation. Subsequently, both surfaces of the leaves were inoculated with a conidial suspension of *C. fulvum.* For curative purposes, the plants were sprayed twice: firstly, 24 h after IOP, and then again 48 h later. The inoculated plants were placed in a humid chamber, maintaining around 90% humidity at 25 °C for two months. At 15 and 30 dpi, disease severity was estimated using the Leaf Doctor application on a smartphone after photographing the primary leaves of each plant against a black background. 

##### Antagonistic Fungi

The experiment comprised five distinct treatments, each replicated four times, following a completely randomized block design. Within each experimental block, nine plants were accommodated, spanning a length of four meters. About two weeks following the emergence of lesions on the tomato leaves that were inoculated with *C. fulvum* (curative strategy), a conidial suspension of each *Trichoderma* spp. isolate was sprayed onto the lesions caused by leaf mold disease. The spore suspension of the necrotrophic mycoparasite *Trichoderma* spp. was generated by gently scraping the surface of a 10 to 15-day-old colony culture. The concentration of the resulting suspension was meticulously adjusted to attain the targeted final concentration of 2 × 10^4^ conidia/mL. The assessment of lesion colonization and its visual scoring was conducted at 15 and 30 dpi.

##### Chemical Fungicide

The study comprised five treatments, each replicated four times, and was organized using a completely randomized block design. Within each experimental block, five plants were used, covering a span of six meters. The in vivo experiment included five treatment groups aimed at assessing the compound’s protective, curative, and post-symptom effects. To evaluate the protective and curative qualities of azoxystrobin, spray applications were administered at intervals of 24, 48, and 96 h before (for protective treatments) and after (for curative treatments) IOP. Control plants were subjected to sprays of sterile tap water 24 h before IOP, while others received treatment with a reference fungicide (Daconil 720 SC). For the assessment of post-symptom activity, certain plants were treated immediately after the initial symptom manifestation, which occurred at 15 dpi. Each concentration of the fungicide constituted a distinct treatment, and four plants were used for each treatment. This experimental setup was replicated twice. Disease-severity measurements were conducted at 22 dpi.

### 4.6. Efficacy Test of Bacterial Strain Candidates for Commercialization as a Biological Product

The in vivo experiment was conducted using two of the five tested bacteria, which exhibited significant inhibition rates in vitro and demonstrated the ability to reduce disease severity in vivo. This was carried out to evaluate their effectiveness under semi-commercial conditions, drawing comparisons with the reference bacteria (*B. subtilis* Y1336 and *P. agglomerans* P10c), which are commercial products already on the market, and the chemical fungicide (azoxystrobin). The protocol for the in vivo test followed the same confrontation procedure as previously described.

### 4.7. FTIR-ATR Analysis

FTIR-ATR analysis was performed to assess the impact of biological treatments on the organic composition of tomato leaves, following the method adapted from Kosa et al. [55]. Fresh tomato leaves were collected from treated and control plants, rinsed gently with distilled water to remove any adhering particles, and air-dried at room temperature. The dried leaves were then cut into small pieces (approximately 1–2 cm^2^) and placed in a desiccator to ensure complete moisture removal. FTIR-ATR spectra were recorded in the wavelength range of 4000–400 cm^−1^ with a spectral resolution of 5 cm^−1^ using a Perkin-Elmer FTIR spectrometer (Perkin-Elmer, Waltham, MA, USA). Each sample was positioned on the ATR crystal using tweezers, and spectra were collected in absorbance mode. To ensure reproducibility, each analysis was repeated three times. The recorded spectra were stored via fiber optics for subsequent analysis. Between measurements, the ATR crystal was cleaned with isopropyl alcohol and lint-free wipes to prevent cross-contamination. The spectra were analyzed to detect characteristic peaks and functional groups, and comparisons were made between treated and control samples to identify changes in chemical composition induced by the biological treatments.

### 4.8. Statistical Analysis

All in vivo and in vitro experiments were conducted twice with replicates, and the resulting datasets were subjected to analysis of variance (ANOVA) using SPSS version 20. When significant effects were detected, means were separated into homogeneous groups using the SNK (Student–Newman–Keuls) and LSD (Least Significant Difference) tests at a significance level of *p* < 0.05.

## 5. Conclusions

This research significantly contributes to the body of knowledge on managing *Cladosporium fulvum*-induced disease in tomato plants. Our study tested bacterial and fungal isolates from the laboratory collection, which had previously shown promising antagonistic effects against various pathogens. Among the five bacterial isolates, *P. agglomerans* ACBP1 was the most effective, achieving results comparable to current commercial products in both in vitro and greenhouse tests. Among the fungal isolates, *Trichoderma* spp. BT3 emerged as the most promising. Additionally, several bacterial isolates produced volatile organic compounds that inhibited the mycelial growth of *C. fulvum*, with *B. amyloliquefaciens* SF14 being the most effective. Further large-scale tests are needed to evaluate the commercial viability of these promising biocontrol agents, which will be considered for commercialization. 

Our findings highlight the remarkable efficacy of azoxystrobin against *C. fulvum*, demonstrating significant protective, curative, and anti-sporulant properties even at low application rates. However, the specific mode of action of strobilurin fungicides, such as azoxystrobin, poses a high risk of rapid resistance development among pathogen populations. Consequently, further research is needed to establish optimal treatment schedules in field conditions to mitigate resistance risk.

Overall, this study provides valuable insights into integrated management strategies for tomato leaf mold disease, suggesting that combining biological control agents with careful fungicide application could offer sustainable and effective disease management.

## Figures and Tables

**Table 1:** Inhibition rate of mycelial growth (%) of *Cladosporium fulvum* obtained by antagonistic bacteria (*Bacillus amyloliquefaciens* SF14, *Alcaligenes faecalis* ACBC1, *Pantoea agglomerans* ACBC2, *P. agglomerans* ACBP1, *P. agglomerans* ACBP2, *Bacillus subtilis* Y1336, and *P. agglomerans* P10c), and azoxystrobin after 5 and 12 days of incubation at 25 °C.

Treatment	5-Day Post-Incubation Period		12-Day Post-Incubation Period	
	Colony Diameter (mm)	Inhibition Rate (%)	Colony Diameter (mm)	Inhibition Rate (%)
Untreated control (Pathogen only)	20.5 ^d^ ± 2.75	0	31.25 ^d^ ± 2.66	0
*Bacillus amyloliquefaciens* SF14	10.25 ^bc^ ± 0.75	50	13.37 ^bc^ ± 1.02	57.2
*Alcaligenes faecalis* ACBC1	13.37 ^c^ ± 1.18	34.8	16.33 ^c^ ± 1.81	47.7
*Pantoea agglomerans* ACBC2	10.5 ^bc^ ± 0.5	48.8	12.38 ^bc^ ± 0.99	60.4
*Pantoea agglomerans* ACBP1	9.87 ^b^ ± 0.37	51.8	11.34 ^b^ ± 0.74	63.7
*Pantoea agglomerans* ACBP2	10.87 ^bc^ ± 0.62	46.9	11.81 ^b^ ± 1.05	62.2
*Bacillus subtilis* Y1336 (Reference bacteria)	9.33 ^b^ ± 0.67	54.4	10.92 ^b^ ± 0.57	65
*Pantoea agglomerans* P10c (Reference bacteria)	9.35 ^b^ ± 0.67	54.4	10.52 ^b^ ± 0.85	66.3
Azoxystrobin (PrioriTop)	0.00 ^a^	100	1.27 ^a^ ± 0.36	95.9

The data (mean ± SD) are the average of two independent trials with four replicates for each treatment. The mean diameters with the same letter in the same column are not significantly different according to the SNK test at *p* < 0.05.

**Table 2:** Inhibition rate of mycelial growth (%) of *Cladosporium fulvum* obtained by sterile filtrates of antagonistic bacteria (*Bacillus amyloliquefaciens* SF14, *Alcaligenes faecalis* ACBC1, *Pantoea agglomerans* ACBC2, *P. agglomerans* ACBP1, *P. agglomerans* ACBP2, *Bacillus subtilis* Y1336, and *P. agglomerans* P10c), after 5 and 12 days of incubation at 25 °C.

Treatment	5-Day Post-Incubation Period		12-Day Post-Incubation Period	
	Colony Diameter (mm)	IR (%)	Colony Diameter (mm)	IR (%)
Untreated control (Pathogen only)	20.5 ^c^ ± 2.75	0	31.25 ^c^ ± 2.66	0
*Bacillus amyloliquefaciens SF14*	9.76 ^a^ ± 0.83	52.4	15.6 ^b^ ± 1.8	50.1
*Alcaligenes faecalis ACBC1*	10.01 ^a^ ± 0.99	51.2	13.3 ^a^ ± 0.65	57.4
*Pantoea agglomerans ACBC2*	15.15 ^b^ ± 1.42	26.1	19.12 ^b^ ± 1.92	38.8
*Pantoea agglomerans ACBP1*	13.37 ^b^ ± 2.15	34.8	16.23 ^b^ ± 1.62	48
*Pantoea agglomerans ACBP2*	10.24 ^a^ ± 0.77	50	14.93 ^b^ ± 1.93	52.2
*Bacillus subtilis Y1336* (Reference bacteria)	9.19 ^a^ ± 0.64	55.2	12.35 ^a^ ± 1.45	60.5
*Pantoea agglomerans P10c* (Reference bacteria)	11.12 ^a^ ± 0.49	45.7	12.91 ^a^ ± 0.95	58.7

The data (mean ± SD) are the average of two independent trials with four replicates for each treatment. The mean diameters with the same letter in the same column are not significantly different according to the SNK test at *p* < 0.05.

**Table 3:** Inhibition rate of mycelial growth (%) of *Cladosporium fulvum* obtained by volatile products secreted by antagonistic bacteria (*Bacillus amyloliquefaciens* SF14, *Alcaligenes faecalis* ACBC1, *Pantoea agglomerans* ACBC2, *P. agglomerans* ACBP1, *P. agglomerans* ACBP2, *Bacillus subtilis* Y1336, and *P. agglomerans* P10c), after 5 and 12 days of incubation at 25 °C.

Treatment	5-Day Post-Incubation Period		12-Day Post-Incubation Period	
	Colony Diameter (mm)	IR (%)	Colony Diameter (mm)	IR (%)
Untreated control (Pathogen only)	20.5 ^c^ ± 2.75	0	31.25 ^c^ ± 2.66	0
*Bacillus amyloliquefaciens SF14*	8.74 ^a^ ± 0.72	57.4	10.17 ^b^ ± 0.41	67.5
*Alcaligenes faecalis ACBC1*	10.26 ^a^ ± 0.76	49.9	11.8 ^b^ ± 0.7	62.2
*Pantoea agglomerans ACBC2*	9.86 ^a^ ± 0.58	51.9	11.7 ^b^ ± 0.7	62.6
*Pantoea agglomerans ACBP1*	10.37 ^a^ ± 0.85	49.4	11.74 ^b^ ± 0.59	62.4
*Pantoea agglomerans ACBP2*	13.74 ^b^ ± 0.93	32.9	17.43 ^a^ ± 1.62	44.2
*Bacillus subtilis Y1336* (Reference bacteria)	9.34 ^a^ ± 0.67	54.4	10.92 ^b^ ± 0.57	65
*Pantoea agglomerans P10c* (Reference bacteria)	9.35 ^a^ ± 0.67	54.4	11.7 ^b^ ± 0.85	62.6

The data (mean ± SD) are the average of two independent trials with four replicates for each treatment. The mean diameters with the same letter in the same column are not significantly different according to the SNK test at *p* < 0.05.

**Table 4:** Inhibition rate of mycelial growth (%) of *Cladosporium fulvum* obtained by antagonistic fungi *Trichoderma* spp. isolated from soils surrounding olive (OT1) and apple (AT2) trees and blueberry plants (BT3), after 5 and 12 days of incubation at 25 °C.

Treatment	5-Day Post-Incubation Period		12-Day Post-Incubation Period	
	Colony Diameter (mm)	IR (%)	Colony Diameter (mm)	IR (%)
Untreated control (Pathogen only)	11.21 ^a^ ± 2.14	-	17.375 ^a^ ± 2.66	-
*Trichoderma* spp. OT1	0.00 ^b^	100	0.00 ^b^	100
*Trichoderma* spp. AT2	0.00 ^b^	100	0.00 ^b^	100
*Trichoderma* spp. BT3	0.00 ^b^	100	0.00 ^b^	100
Azoxystrobin (PrioriTop)	0.00 ^b^	100	1.27 ^b^ ± 0.36	95.9

The data (mean ± SD) are the average of two independent trials with four replicates for each treatment. The mean diameters with the same letter in the same column are not significantly different according to the SNK test at *p* < 0.05.

**Table 5:** Inhibition rate of mycelial growth (%) of *Cladosporium fulvum* obtained by the fungicide azoxystrobin, tested at 5 (D5), 10 (D10), and 20 µg a.i.mL^−1^ (D20), after 5 and 12 days of incubation at 25 °C. The standard fungicide, chlorothalonil, was tested at 100 µg a.i.mL^−1^ (D100).

Treatment	5-Day Post-Incubation Period		12-Day Post-Incubation Period	
	Colony Diameter (mm)	IR (%)	Colony Diameter (mm)	IR (%)
Untreated control (Pathogen only)	21.83 ^c^ ± 2.11	---	30.94 ^c^ ± 3.34	---
Azoxystrobin D5	1.92 ^b^ ± 0.16	91.2	2.56 ^b^ ± 0.17	91.7
Azoxystrobin D10	1.65 ^b^ ± 0.23	92.4	1.95 ^b^ ± 0.11	93.7
Azoxystrobin D20	0.6 ^a^ ± 0.26	97.2	0.88 ^a^ ± 0.21	97.1
Chlorothalonil D100 (Standard fungicide)	0.36 ^a^ ± 0.28	98.3	1.36 ^b^ ± 0.24	95.6

The data (mean ± SD) are the average of two independent trials with four replicates for each treatment. The mean diameters with the same letter in the same column are not significantly different according to the SNK test at *p* < 0.05.

**Table 6:** Greenhouse trial efficacies (incidence (I) and severity (S) reduction compared to the control plants) obtained with three selected antagonistic fungi and a commercial fungicide against tomato leaf mold disease. Incidence and severity were recorded during the 30-day post-incubation period.

Treatments	Incidence (%)	I. Control (%)	Severity (%)	S. Control (%)
Untreated control (Pathogen only)	94 ^a^	0	31.7 ^a^	0
*Trichoderma* spp. OT1	10 ^b^	89.4	0.6 ^c^	98.2
*Trichoderma* spp. AT2	8 ^b^	91.5	0.3 ^c^	99.1
*Trichoderma* spp. BT3	3 ^b^	96.8	0.1 ^c^	99.7
Azoxystrobin (PrioriTop)	3 ^b^	96.8	7 ^b^	77.9

Values of incidence and severity with the same letter (a–c) were not significantly different according to the SNK test (*p* ≤ 0.05).

**Table 7:** Disease severity and control efficacy (%) of tomato leaf mold caused by *C. fulvum* after applications of azoxystrobin and chlorothalonil 24, 48, and 96 h before inoculation of plants by the pathogen (protective activity).

Treatments	Hours before Pathogen Inoculation					
	96		48		24	
	Number of Lesions *	Control (%) **	Number of Lesions	Control (%)	Number of Lesions	Control (%)
Untreated control (Pathogen only)	20.45 ^d^	---	20.45 ^c^	---	20.45 ^d^	---
Azoxystrobin D5	6.16 ^c^	70.1	3.00 ^b^	85.30	2.45 ^c^	88
Azoxystrobin D10	2.86 ^b^	86	1.84 ^a^	91.00	1.22 ^b^	94
Azoxystrobin D20	1.22 ^a^	94	0.67 ^a^	96.70	0.67 ^a^	96.7
Chlorothalonil D100	3.06 ^b^	85	0.94 ^a^	95.40	0.61 ^a^	97

Tested concentrations: 5 (D5), 10 (D10), 20 (D20), and 100 (D100) µg a.i.mL^−1^. * Disease severity was determined based on the number of lesions on 45 randomly selected leaves per treatment. ** Percentage control values were calculated as 100 − severity (treated)/severity (untreated). Means followed by the same letters in the column are not significantly different according to the SNK test at *p* = 0.05.

**Table 8:** Disease severity and control efficacy (%) of tomato leaf mold caused by *C. fulvum* after applications of azoxystrobin and chlorothalonil 24, 48, and 96 h after inoculation of plants by the pathogen (Curative activity).

Treatment	Hours after Pathogen Inoculation					
	24		48		96	
	Number of Lesions *	Control (%) **	Number of Lesions	Control (%)	Number of Lesions	Control (%)
Untreated control (Pathogen only)	22.37 ^d^	---	22.37 ^d^	---	22.37 ^c^	---
Azoxystrobin D5	6.75 ^c^	69.8	8.83 ^c^	60.50	12.3 ^b^	45
Azoxystrobin D10	2.46 ^b^	89	5.27 ^b^	76.40	8.94 ^a^	60
Azoxystrobin D20	1.34 ^a^	94	1.2 ^a^	94.60	7.49 ^a^	66.5
Chlorothalonil D100	2.01 ^b^	91	5.74 ^b^	74.30	11.63 ^b^	48

Tested concentrations: 5 (D5), 10 (D10), 20 (D20), and 100 (D100) µg a.i.mL^−1^. * Disease severity was determined based on the number of lesions on 45 randomly selected leaves per treatment. ** Percentage control values were calculated as 100 − severity (treated)/severity (untreated). Means followed by the same letters in the column are not significantly different according to the SNK test at *p* = 0.05.

**Table 9:** Spore production of *C. fulvum* on tomato leaves after post-symptom applications of azoxystrobin and chlorothalonil fungicides (post-symptom activity).

Treatments	Number of Spores *	Sporulation Inhibition (%) **
Untreated control (Pathogen only)	8.6 ^d^	---
Azoxystrobin D5	6.53 ^c^	24
Azoxystrobin D10	3.84 ^b^	55.3
Azoxystrobin D20	1.06 ^a^	87.6
Chlorothalonil D100	4.71 ^b^	45.2

Tested concentrations: 5 (D5), 10 (D10), 20 (D20), and 100 (D100) µg a.i.mL^−1^. * Number of spores (×10^3^) per cm^2^ diseased leaf surface. ** Percentage of sporulation inhibition values were calculated as 100—sporulation (treated)/sporulation (untreated). Means followed by the same letters in the column are not significantly different according to the SNK test at *p* = 0.05.

**Table 10:** General band assignments of the FTIR spectra of the different treatment of leaves of tomato plants used in all in vivo trials.

Wavenumber (cm^−1^)	Biomarker
1737	Pectin
1655	Protein (Amide I)
1615–1590	Lignin
1547	Protein (Amide II)
1515–1505	Lignin
1372	Cellulose
1245	Hemicellulose
1161	Cellulose
1060	Cellulose
930–800	Β-glycosidic linkages

**Table 11:** Integrated absorption bands in leaves’ FTIR spectra of the treated and untreated tomato plants.

Absorption Bands (cm^−1^)								
	Pectin	Protein (Amid I)		Lignin		Hemicellulose	Cellulose	Protein (Amid II)
	1760–1720	1710–1620	1615–1590	1480–1455	1445–1410	1261–1200	1090–1022	1520–1560
ACBC1	0.71 ^ab^ ± 0.14	0.85 ^a^ ± 0.15	1.63 ^b^ ± 0.3	0.73 ^a^ ± 0.11	0.00 ^a^	0.85 ^a^ ± 0.09	2.55 ^b^ ± 0.17	0.83 ^a^ ± 0.09
ACBC2	0.77 ^ab^ ± 0.11	1.93 ^ab^ ± 0.2	2.07 ^b^ ± 0.14	0.00 ^a^	0.00 ^a^	0.94 ^a^ ± 0.09	3 ^b^ ± 0.19	0.00 ^a^
ACBP1	0.38 ^a^ ± 0.12	0.95 ^a^ ± 0.12	1.09 ^b^ ± 0.16	0.43 ^a^ ± 0.13	0.58 ^a^ ± 0.08	0.44 ^a^ ± 0.07	1.55 ^ab^ ± 0.13	0.55 ^a^ ± 0.04
ACBP2	0.31 ^a^ ± 0.1	0.35 ^a^ ± 0.12	0.56 ^a^ ± 0.12	0.43 ^a^ ± 0.12	0.58 ^a^ ± 0.08	0.42 ^a^ ± 0.07	1.56 ^ab^ ± 0.012	0.52 ^a^ ± 0.04
SF14	0.54 ^ab^ ± 0.02	0.54 ^a^ ± 0.13	1.42 ^b^ ± 0.22	0.7 ^a^ ± 0.09	0.00 ^a^	0.68 ^a^ ± 0.08	2.25 ^b^ ± 0.16	0.84 ^a^ ± 0.08
Y1336	1.72 ^b^ ± 0.45	3.71 ^b^ ± 0.14	3.82 ^c^ ± 0.45	1.62 ^b^ ± 0.12	2.37 ^b^ ± 0.11	1.87 ^b^ ± 0.11	6.31 ^c^ ± 0.9	1.98 ^b^ ± 0.11
P10c	0.993 ^b^ ± 0.09	9.7 ^c^ ± 0.21	2.1 ^b^ ± 0.13	0.87 ^a^ ± 0.09	0.00 ^a^	1.02 ^ab^ ± 0.1	2.86 ^b^ ± 0.3	1.1 ^b^ ± 0.12
OT1	0.00 ^a^	1.6 ^ab^ ± 0.13	1.48 ^b^ ± 0.24	0.8 ^a^ ± 0.09	1.06 ^b^ ± 0.13	0.86 ^a^ ± 0.09	3.01 ^b^ ± 0.19	1.01 ^b^ ± 0.12
AT2	0.00 ^a^	2.06 ^ab^ ± 0.15	2.27 ^b^ ± 0.12	1.02 ^b^ ± 0.11	1.41 ^b^ ± 0.12	1.14 ^b^ ± 0.1	0.00 ^a^	1.24 ^b^ ± 0.12
BT3	0.00 ^a^	2.04 ^ab^ ± 0.15	2.03 ^b^ ± 0.14	7.9 ^c^ ± 0.14	1.04 ^b^ ± 0.11	0.83 ^a^ ± 0.09	0.00 ^a^	1.07 ^b^ ± 0.12
Azoxystrobin	0.00 ^a^	0.77 ^a^ ± 0.11	2.07 ^b^ ± 0.15	0.00 ^a^	0.00 ^a^	0.94 ^a^ ± 0.09	3 ^b^ ± 0.19	0.00 ^a^
Control	5.31 ^c^ ± 0.05	9.77 ^c^ ± 0.14	4.07 ^c^ ± 0.16	0.817 ^a^ ± 0.09	0.56 ^a^ ± 0.08	0.93 ^a^ ± 0.09	5.33 ^c^ ± 0.4	0.99 ^a^ ± 0.09

1737 (1760–1720 cm^−1^); 1686 (1710–1620 cm^−1^); 1606 (1615–1590 cm^−1^); 1463 (1480–1455 cm^−1^); 1426 (1445–1410 cm^−1^); 1210 (1261–1200 cm^−1^); 1066 (1090–1022 cm^−1^); 1535 (1520–1560 cm^−1^). Means in the same column followed by the same letter are not significantly different according to the LSD test *p* ≤ 0.05.

## Data Availability

The data that support the findings of this study are available from the corresponding author upon reasonable request.

## Supplementary Materials

The following supporting information can be downloaded at: https://www.mdpi.com/article/10.3390/plants13162213/s1, Figure S1. In vitro confrontation test revealing antagonistic activity of bacterial isolates against *C. fulvum* on PDA medium after 12 days of incubation at 25 °C. (a) Untreated control (pathogen only; *C. fulvum*); (b) Azoxystrobin fungicide; (c) *Bacillus amyloliquefaciens* SF14; (d) *Pantoea agglomerans* ACBP1; (e) *Pantoea agglomerans* ACBP2; (f) *Pantoea agglomerans* ACBC2; (g) *Bacillus subtilis* Y1336 (reference bacteria); (h) *Alcaligenes faecalis* ACBC1; (i) *Pantoea agglomerans* P10c (reference bacteria). Figure S2. In vitro confrontation test revealing antagonistic activity of *Trichoderma* spp. isolates against *C. fulvum* on PDA medium after 12 days of incubation at 25 °C. (a) Treatment with *Trichoderma* spp. isolate from soil surrounding olive trees (OT1); (b) Treatment with *Trichoderma* spp. isolate from the soil surrounding apple trees (AT2); (c) Treatment with *Trichoderma* spp. isolate from the soil surrounding blueberry plants (BT3); (d) Untreated control (pathogen only; *C. fulvum*). Figure S3. In vivo confrontation test revealing antagonistic activity of *Trichoderma* spp. isolates against *C. fulvum* on tomato leaves after 30 days of incubation at 25 °C and 85% RH. (A1) and (A2): adaxial and abaxial faces of leaves treated with azoxystrobin fungicide and control leaf (pathogen only, *C. fulvum*); (B1) and (B2): adaxial and abaxial faces of leaves treated with *Trichoderma* spp. isolate (OT1); (C1) and (C2): adaxial and abaxial faces of leaves treated with *Trichoderma* spp. isolate (AT2); (D1) and (D2): adaxial and abaxial faces of leaves treated with *Trichoderma* spp. isolate (BT3). Figure S4. Pure colonies of the three isolates of *Trichoderma* spp. used in this study grown on a PDA medium. (a) The OT1 isolate was obtained from the soil surrounding olive trees; (b) The AT2 isolate was obtained from the soil surrounding apple trees; (c) The BT3 isolate was obtained from the soil surrounding blueberry plants.
