# Supplementary material for: Exploring the Antifungal Activity of Moroccan Bacterial and Fungal Isolates and a Strobilurin Fungicide in the Control of Cladosporium fulvum, the Causal Agent of Tomato Leaf Mold Disease
Source: Plants (Basel). 2024 Aug 9;13(16):2213. doi: 10.3390/plants13162213 (PMC11360235; doi:10.3390/plants13162213)
Supplement: Supplementary file 1 [file plants-13-02213-s001.zip › plants-3121558-supplementary.pdf]

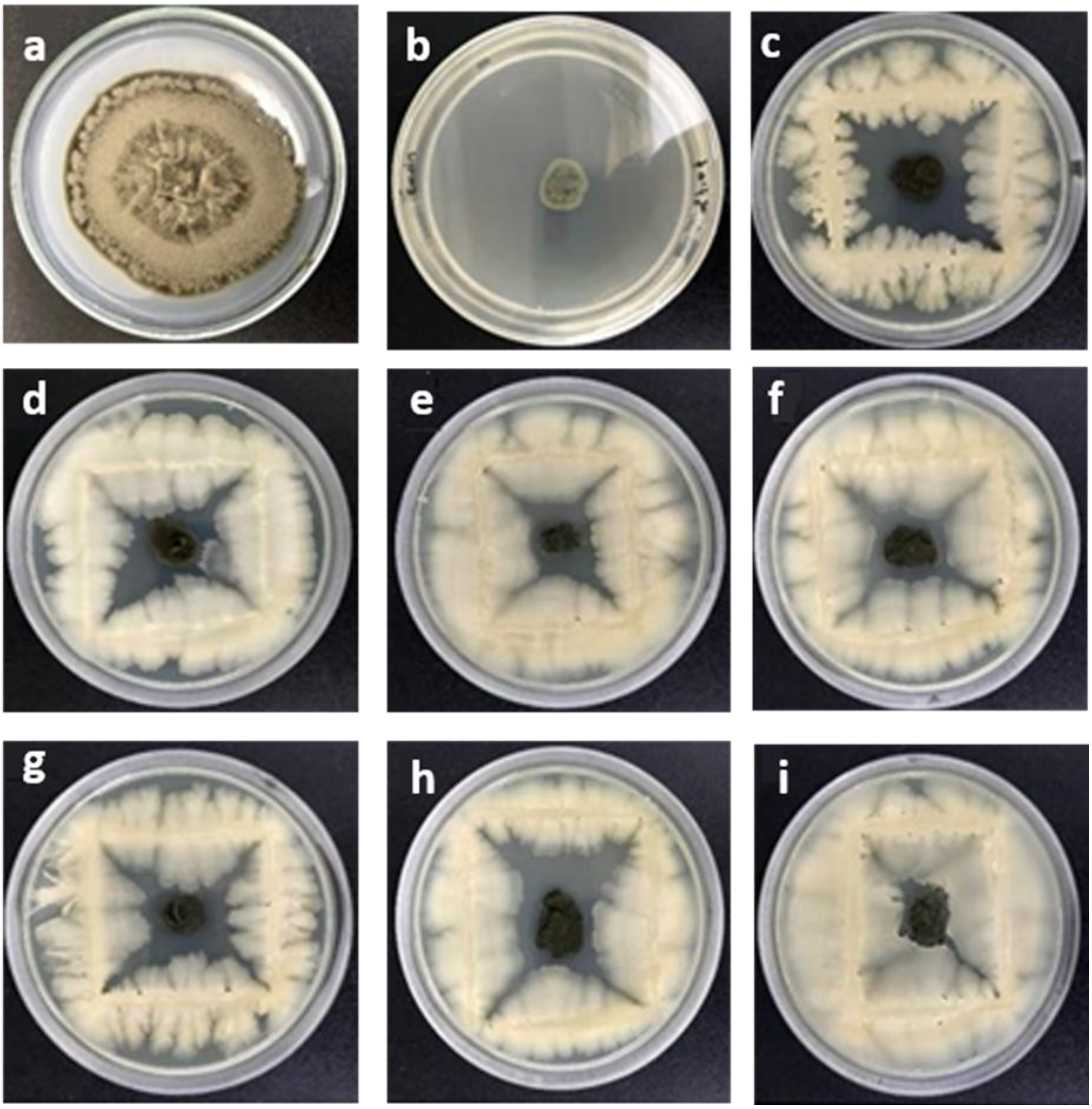

Figure S1: *In vitro* confrontation test revealing antagonistic activity of bacterial isolates against *C. fulvum* on PDA medium after 12 days of incubation at 25°C. (a) Untreated control (pathogen only; *C. fulvum*); (b) Azoxystrobin fungicide; (c) *Bacillus amyloliquefaciens* SF14; (d) *Pantoea agglomerans* ACBP1; (e) *Pantoea agglomerans* ACBP2; (f) *Pantoea agglomerans* ACBC2; (g) *Bacillus subtilis* Y1336 (reference bacteria); (h) *Alcaligenes faecalis* ACBC1; (i) *Pantoea agglomerans* P10c (reference bacteria).

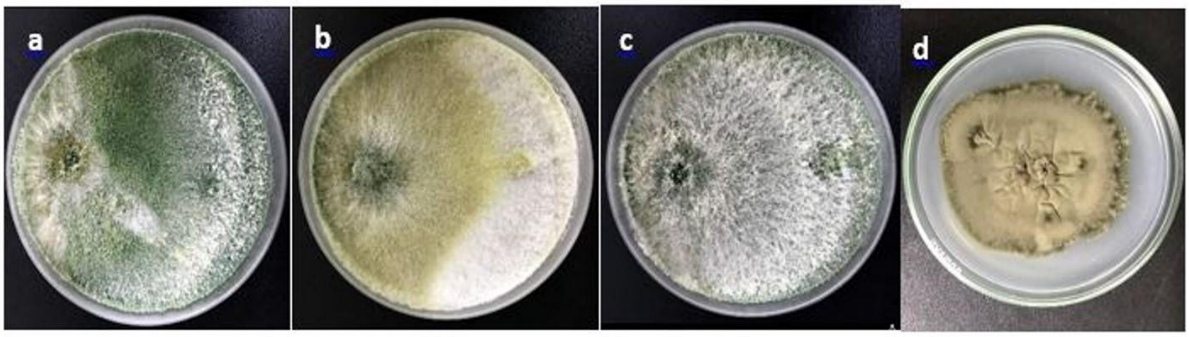

Figure S2. *In vitro* confrontation test revealing antagonistic activity of *Trichoderma* spp. isolates against *C. fulvum* on PDA medium after 12 days of incubation at 25°C. (a) Treatment with *Trichoderma* spp. isolate from soil surrounding olive trees (OT1); (b) Treatment with *Trichoderma* spp. isolate from the soil surrounding apple trees (AT2); (c) Treatment with *Trichoderma* spp. isolate from the soil surrounding blueberry plants (BT3); (d) Untreated control (pathogen only; *C. fulvum*).

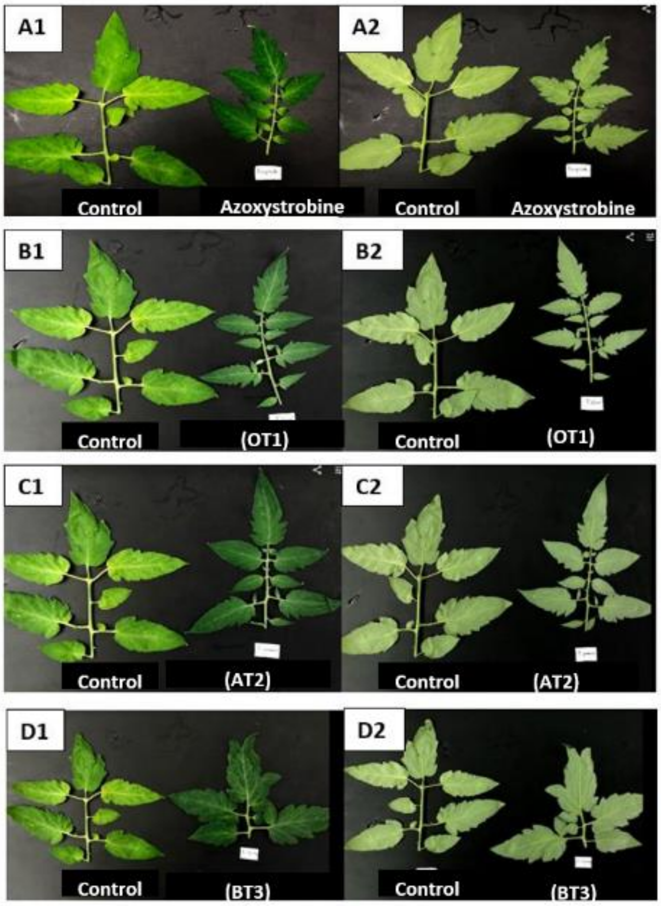

Figure S3. *In vivo* confrontation test revealing antagonistic activity of *Trichoderma* spp. isolates against *C. fulvum* on tomato leaves after 30 days of incubation at 25°C and 85% RH. (A1) and (A2): adaxial and abaxial faces of leaves treated with azoxystrobin fungicide and control leaf (pathogen only, *C. fulvum*); (B1) and (B2): adaxial and abaxial faces of leaves treated with *Trichoderma* spp. isolate (OT1); (C1) and (C2): adaxial and abaxial faces of leaves treated with *Trichoderma* spp. isolate (AT2); (D1) and (D2): adaxial and abaxial faces of leaves treated with *Trichoderma* spp. isolate (BT3).

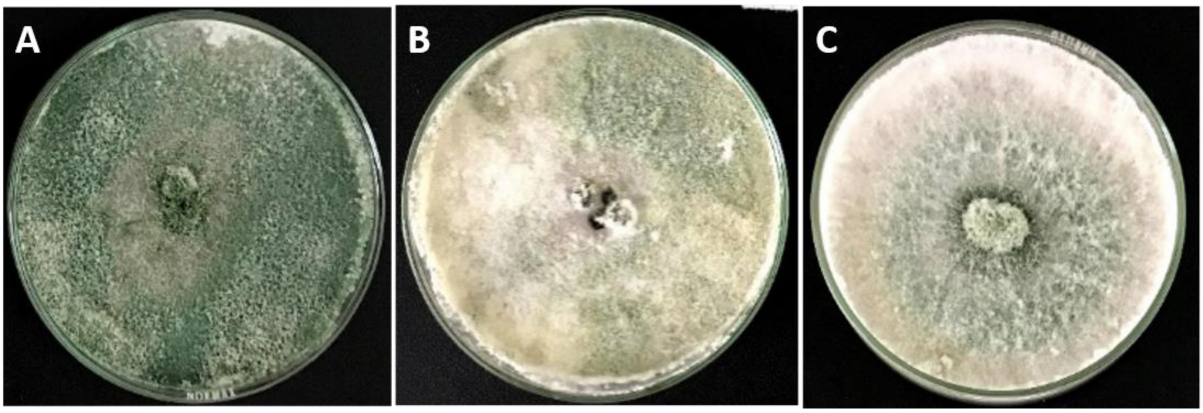

Figure S4. Pure colonies of the three isolates of *Trichoderma* spp. used in this study grown on a PDA medium. **(a)** The OT1 isolate was obtained from the soil surrounding olive trees; **(b)** The AT2 isolate was obtained from the soil surrounding apple trees; **(c)** The BT3 isolate was obtained from the soil surrounding blueberry plants.
